# A Review of Methods for Sleep Arousal Detection Using Polysomnographic Signals

**DOI:** 10.3390/brainsci11101274

**Published:** 2021-09-26

**Authors:** Xiangyu Qian, Ye Qiu, Qingzu He, Yuer Lu, Hai Lin, Fei Xu, Fangfang Zhu, Zhilong Liu, Xiang Li, Yuping Cao, Jianwei Shuai

**Affiliations:** 1 Department of Physics, and Fujian Provincial Key Laboratory for Soft Functional Materials Research, Xiamen University, Xiamen 361005, China; 19820181154034@stu.xmu.edu.cn (X.Q.); qiuye@stu.xmu.edu.cn (Y.Q.); qingzuhe@stu.xmu.edu.cn (Q.H.); 19820191153015@stu.xmu.edu.cn (Y.L.); linhaiphysics@gmail.com (H.L.); xmuxu@stu.xmu.edu.cn (F.X.); 21620190154561@stu.xmu.edu.cn (F.Z.); liuzhilong@stu.xmu.edu.cn (Z.L.); xianglibp@xmu.edu.cn (X.L.); 2 Department of Psychiatry of Second Xiangya Hospital, Central South University, Changsha 410011, China; 3 National Institute for Data Science in Health and Medicine, and State Key Laboratory of Cellular Stress Biology, Innovation Center for Cell Signaling Network, Xiamen University, Xiamen 361102, China; 4 Wenzhou Institute, University of Chinese Academy of Sciences, Wenzhou 325001, China; 5 Oujiang Laboratory (Zhejiang Lab for Regenerative Medicine, Vision and Brain Health), Wenzhou 325001, China

**Keywords:** sleep arousal, polysomnography (PSG), machine learning, deep learning

## Abstract

Multiple types of sleep arousal account for a large proportion of the causes of sleep disorders. The detection of sleep arousals is very important for diagnosing sleep disorders and reducing the risk of further complications including heart disease and cognitive impairment. Sleep arousal scoring is manually completed by sleep experts by checking the recordings of several periods of sleep polysomnography (PSG), which is a time-consuming and tedious work. Therefore, the development of efficient, fast, and reliable automatic sleep arousal detection system from PSG may provide powerful help for clinicians. This paper reviews the automatic arousal detection methods in recent years, which are based on statistical rules and deep learning methods. For statistical detection methods, three important processes are typically involved, including preprocessing, feature extraction and classifier selection. For deep learning methods, different models are discussed by now, including convolution neural network (CNN), recurrent neural network (RNN), long-term and short-term memory neural network (LSTM), residual neural network (ResNet), and the combinations of these neural networks. The prediction results of these neural network models are close to the judgments of human experts, and these methods have shown robust generalization capabilities on different data sets. Therefore, we conclude that the deep neural network will be the main research method of automatic arousal detection in the future.

## 1. Introduction

The appearance of sleep arousals (also known as microarousals) reflects the interruption and fragmentation of sleep and is a harbinger of the presence of somnipathy. Frequent microarousals can cause sleep disruption, sleep fragmentation, sleep disorder, aggravating daytime sleepiness, and other symptoms [1]. An increasing amount of evidence indicates that sleep arousals diseases are the concomitant symptoms of other diseases, including weight gain, depression, heart diseases, and diabetes. Therefore, advancing our current understanding of microarousals neurophysiology is not only a challenging research issue but also a public health issue.

Microarousals can also be spontaneous, caused by grinding teeth, partial airway obstruction, or even snoring [2]. A certain amount of spontaneous arousals seems to be an intrinsic part of physiological sleep [3,4], but excessive arousals can disrupt healthy sleep.

Polysomnography (PSG) collects all of the vital signs in a multidimensional time series. The vital signs include electroencephalogram (EEG), electromyography (EMG), electrocardiography (ECG), electrooculography (EOG), blood oxygen saturation level (SaO_2_), respiratory airflow (airflow), and respiratory movement (chest ABD). Normal and abnormal brain activities are typically picked up by EEG. Some neuropathic disorders leave their signature on EEG [5,6,7]. PSG is the gold standard for detecting sleep disorders.

The physiological band of interest for PSG signals usually ranges from 0.01 to several hundred cycles per second. The lowest band in conventional EEG studies has a lower limit of 0.5 Hz or 1.0 Hz as the ‘slow frequency’ and ‘sub-slow’ EEG bands, while 100 Hz corresponds to the highest frequency of the EEG band [8,9]. The ECG spectrum is generally considered to be 0.05–100 Hz [10]. Jarvis et al. [11] suggested that ECG frequency associated with sleep apnea can be reduced to 0.02 Hz. EMG ranges from 5.0 Hz to higher frequencies up to 450 Hz [12]. Respiration movements, airflow, and other forms of SaO_2_ are low-frequency phenomena with activity ranging from 0.05 Hz to 0.35 Hz [13].

The paper is organized as follows: in Section 1, we discuss the hazards of arousals, arousal detection, and the range of PSG signals. In Section 2, we introduce the clinical medical background of arousal, arousal indicators in the American Academy of Sleep Medicine (AASM), and the cyclic alternating pattern (CAP). The criteria for selecting articles are given in Section 3. In Section 4, we introduce three public datasets and some local datasets appearing in the article and discuss the different measurement indicators. In Section 5, the classification methods based on traditional machine learning are discussed. In Section 6, we present the methods using deep learning. In Section 7, some commercial products of arousal detection are introduced. In Section 8, we review some methods for CAP detection. The paper is concluded in Section 9.

## 2. Microarousal Events

In 2004, the American Academy of Sleep Medicine (AASM) produced a manual of new sleep staging rules, updating criteria for staging sleep, as well as for staging wake, respiratory, cardiac, and motor events. Accordingly, microarousals are defined as the transition from sleep to wakefulness, or from rapid eye movement (REM) to non-rapid eye movement. Microarousals of the brain are an abrupt shift in EEG frequency including alpha, theta and/or frequencies greater than 16 Hz (but excluding the spindle band) which typically last for 3–15 s and last at least 3 s, but not including the spindle band [14].

The microarousal does not mean complete awakening from sleep but partial “arousal” from slow-wave sleep, which can happen at any stage of sleep. Sudden changes in brain wave activity patterns are an important feature of microarousals [15]. Microarousals are usually found in non-rapid eye movement phase II (N2) or REM phase based on conventional scores [16,17], and they also occur during REM phase with an increase in EMG signal [18,19].

**Figure 1:**
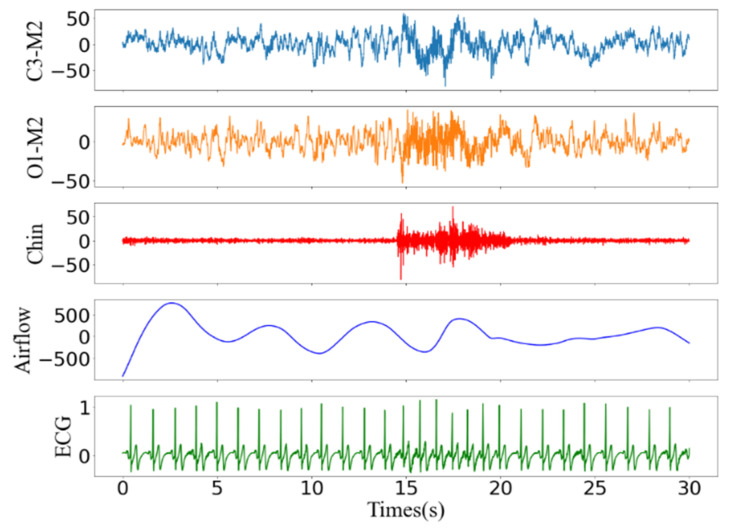
EEG channel diagram during arousal event.

**Figure 2:**
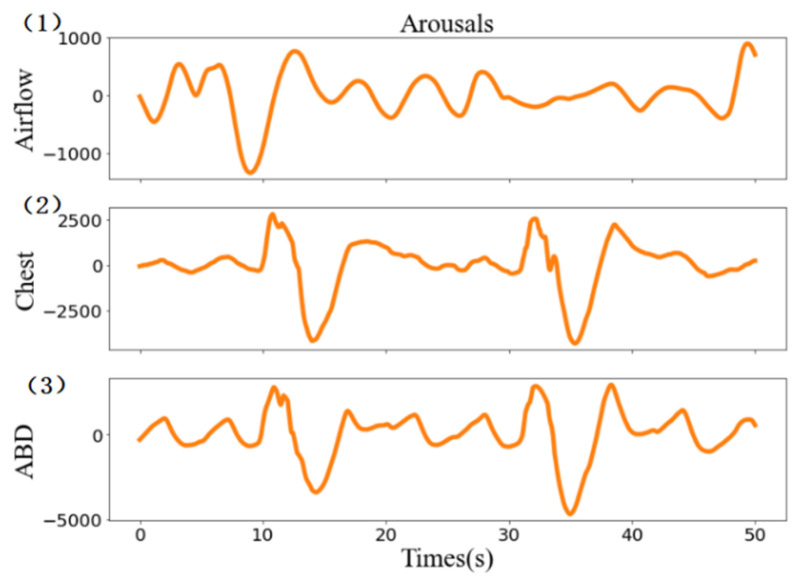
Airflow, chest, and ABD channels during awakening events.

Microarousal can cause PSG signals to present different fluctuations around the normal activity. Screening for sleep disorders is aided by several signals or even a single signal [20], such as ECG [21], pulse oximeter [22], breathing [23], snoring [24], or nasal airway pressure [23]. The main signs of micro awakening in the new AASM manual [25], as examples shown in Figure 1, are as follows:

(1) EEG channel: sleep lasts for at least 10 s, followed by a sudden change in EEG frequency for at least 3 s.
(2) EMG channel: amplitude increases [26], and the degree of increase is related to sleep stage.
(3) ECG channel: this is related to the increase in cardiac activity [27], and the increase in the heart rate depends on the degree of arousal.
(4) Respiratory related channels: includes chest, airflow and abdominal (ABD), as shown as shown in Figure 2, the respiratory sequence of the channel changes continuously for more than 10 s, which is manifested in the increase in the respiratory effort or the flattening of the inspiratory part of the respiratory channel.

As an extension of PSG technology, the CAP [28] is a sufficient supplement to evaluate sleep quality and to detect arousal states. The CAP is a periodic EEG activity occurring during NREM sleep. A CAP cycle is defined as a phase A period and the following phase B period. B period separates two successive phase A periods with an interval lasting a minute or less. At least two CAP cycles are required to form a CAP sequence [28].

Phase A periods are subdivided into three subtypes [28,29], i.e., subtype A1, A2, and A3. Subtype A2 and A3 mark the arousal of the central nervous system.

Subtype A1 is an important component of NREM sleep. Its frequency range is generally between 0.25 Hz and 2.5 Hz [29], and its origin is located in the frontal lobe, which plays a protective role in maintaining the continuity of sleep.

Subtypes A2 and A3 are generally considered to be the prelude to REM sleep [29]. Their origin is located in the parietal and occipital parts [29], which has the function of maintaining sleep.

Phase B consisted of the background rhythm [29] to that stage.

The three subtypes of phase A include NREM sleep arousal (A2 and A3) and the sleep maintenance and protection process (A1). The general indicators used for CAP analysis are the total occurrence rate of CAP (the proportion of the CAP sequence in the whole NREM sleep); the total number; or the percentage of A1, A2, and A3 subtypes. Generally, the increase in CAP rate is a significant feature of the decrease in sleep quality [28].

CAP analysis cannot replace the traditional analysis methods, but it can further our understanding of the microstructure of human sleep as a supplementary means. For the integrity of the subject content, the CAP methods are introduced below.

Increased amounts of CAP are often observed in sleep-disordered breathing (SDB) and insomnia [28,30], sleep movement disorders (periodic leg movements (PLM) [28,31], restless leg syndrome (RLS) [28,32]), parasomnias such as REM behavior disorder (RBD), epileptic diseases such as nocturnal frontal lobe epilepsy (NFLE) [28,33], and hypersomnia of central origin such as narcolepsy [28,34].

## 3. Materials and Methods

**Figure 3:**
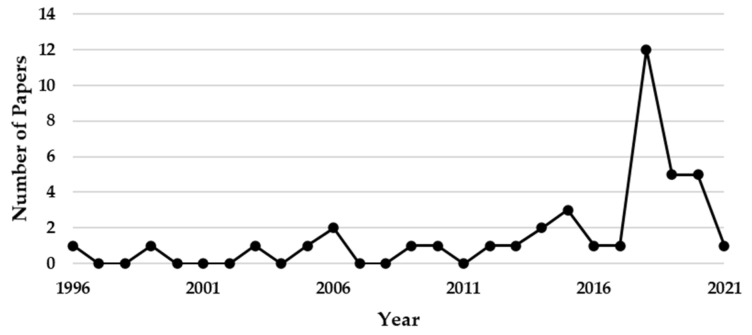
Number of publications papers which met the inclusion criteria per year.

We reviewed the literature using the ResearchGate, Springer, and IEEE databases from 1980 to 2021 for this review paper. Each study in this review was screened to meet all the inclusion criteria. The inclusion criteria are (A) human studies, (B) studies published in English or Chinese, and (C) experimental studies using a quantitative approach. The criteria of article exclusion are given as follows: (A) articles that do not address the phrase used for search, which was completed by reviewing the abstracts and results; (B) articles that are not research-type papers, e.g., review papers, editorials, case studies, and conference abstracts; and (C) articles that do not have a definite data source, a reproducible model structure, or specific experimental results. We used the following search terms: (“sleep arousal” or “microarousal” [title]) AND (“detection” OR “algorithm” OR “method” OR “model” [title/abstract]). For the CAP detection, we used the following search terms: (“Cyclic Alternating Pattern” or “CAP” [title]) AND (“detection” OR “algorithm” OR “method” OR “model” [title/abstract]). This search produced 304 articles. Finally, 40 of them met the inclusion criteria after full-text review and are included in this review paper. The number of publications per year is provided in Figure 3.

## 4. Introduction of Public PSG Data Sets

At present, most related approaches are usually limited to relatively small (less than 40 recordings) and private datasets. Whether the detection ability of these algorithms can be extended to larger samples or different databases remains a problem. At the same time, we are not sure that these algorithms perform well in a clinical (uncontrolled) environment.

On the other hand, different databases involve different signal acquisition and digitization methods, different population characteristics and different expert interpretations. Even if the arousal scoring is limited to the same recordings, human subjectivity will still lead to differences.

**Figure 4:**
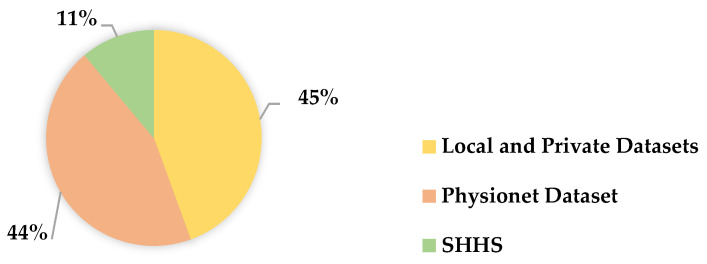
Percentage distribution of different databases.

Therefore, we need to build a large and heterogeneous database to verify the generalization ability of sleep arousal detection methods and promote these methods to be applied in clinical practice. In this review, we introduce three external and publicly accessible sources, namely the PhysioNet dataset, Wisconsin Sleep Cohort dataset, and Sleep Heart Health Study (SHHS) dataset. Each of the databases is described in detail below, Figure 4 shows the percentage of the studies that used different databases.

### 4.1. 2018 PhysioNet/Computing in Cardiology Challenge

In the 2018 PhysioNet/Computing in Cardiology Challenge [2], the PhysioNet dataset consisted of 1985 subjects with sleep disorders who were monitored at the Massachusetts General Hospital (MGH) Sleep Laboratory. For each subject, 13 different physiological signals were collected during the PSG sleep study and manually scored by a certified sleep technician at the MGH Sleep Laboratory according to the AASM guidelines. The 13 different physiological signals includes EEG of six channels (F3-M2, F4-M1, C3-M2, C4-M1, O1-M2, and O2-M1), left eye EOG, EMG lead (Chin1-2) placed under the chin, respiratory movements in the chest and abdomen (chest and abdomen), ECG, SaO_2_, and single lead of airflow [2].

The publicly available training sets include 994 PSG recordings associated with sleep, respiratory events and arousal annotations. The annotations of the other 989 PSG recordings are kept hidden by the Challenge organizers as test sets. The labels of sleep stages include wakefulness; REM sleep; stages 1, 2, and 3 of non-REM sleep; and the undefined stage. Annotated arousals are classified as spontaneous arousal, respiratory effort-related arousal (RERA), bruxism, hypoventilation, apnea (central, obstructive, and mixed), vocalization, snoring, periodic leg movements, Cheyne–Stokes respiration, or partial airway obstruction [2]. The target values for the non-apnea arousals, the apnea (hypopnea)-arousals and the non-arousals sections are set to ‘−1’, ‘1’, and ‘0’, respectively. More details of the PhysioNet dataset can be found in the literature [2].

### 4.2. Wisconsin Sleep Cohort (WSC)

WSC is a local population-based study. Some participants suffer from sleep disorders ranging from normal to severe. WSC collects information such as personal sleep patterns, quality, time, and related disorders. According to AASM criteria, two medical professionals annotated arousals, sleep stages, leg movement events, and respiratory events.

Qualified investigators and organizations can obtain data access rights by completing the application form on the official website at https://show.wisc.edu/ (accessed on 11 August 2021).

### 4.3. Sleep Heart Health Study (SHHS)

Granted by the Case Western Reserve University, SHHS was developed by the National Heart Lung and Blood Institute to determine cardiovascular and other consequences of sleep-disordered breathing [35]. SHHS is regarded as a resource for subsequent studies related to sleep disorders. There are 10,000 nightly PSG records of 79,456 h of clinical data. These data have been recorded in clinical sleep laboratories for more than eight years and are highly robust to physical variability among patients. The SHHS dataset was scored using the Rechtschaffen and Kales (R&K) guidelines. SHHS’ data are available at https://www.sleepdata.org/ (accessed on 11 August 2021).

### 4.4. The CAP Sleep Database

The CAP Sleep Database [28,34] is a collection of 108 polysomnographic recordings registered at the Sleep Disorders Center of the Ospedale Maggiore of Parma, Italy. The included subjects were healthy people who did not present any neurological disorders and were free of drugs affecting the central nervous system, and patients diagnosed with NFLE, RBD, PLM, insomniac, narcoleptic, SDB, or bruxism. The labels have the following fields [28,34]:

(1) Sleep stage (W = wake, S1–S4 = sleep stages, R = REM, MT = body movements);
(2) Body position (left, right, prone, or supine; not recorded in some subjects);
(3) Time of day (hh:mm:ss);
(4) Event (either a sleep stage (SLEEP-S0 … S4, REM, MT), or phase A of CAP);
(5) Duration (in seconds);
(6) Location (the signal(s) in which the event can be observed).

### 4.5. Comprehensive Comparison of Database

The properties such as, the location of the participants, sex ratio, age, and medical history are discussed for the three public datasets and some local datasets in Table 1.

First, most datasets regard adult subjects. Second, many datasets have the problem of an insufficient sample size. Third, less than half of the datasets distinguish the types of sleep arousals. Fourth, most datasets have a balanced proportion of men and women, but few datasets regard a specific type of patient, such as the dataset for studying the sleep of elderly men with osteoporosis. Fifth, some datasets regard patients with one or several diseases, such as patients with cardiovascular disease or stress drivers. Sixth, there is no work that examines the differences between races among various automatic arousal recognition methods. Finally, the numbers of arousal events are much smaller than those of non-arousal events in most datasets.

### 4.6. Discussion of Different Measurement Indicators

Accuracy represents the proportion of correct judgment of the model in the total number. Although accuracy can judge the overall accuracy rate, when the samples are unbalanced, for example, when there are 90 positive samples and 10 negative samples in the sample set, the model only needs to predict all samples as positive samples, then the model can achieve 90% accuracy. Therefore, in the case of unbalanced samples, the high accuracy obtained is meaningless.

Precision (specificity) refers to the probability that all samples predicted to be positive are actually positive samples. Precision represents the prediction accuracy in the positive sample results.

Correspondingly, the recall rate (sensitivity) measures the recognition ability of the classifier.

In medical clinical practice, relatively sensitivity of the model is desirable to doctors, because if a patient suffers from a disease that is not detected by the disease detection instrument, the patient’s treatment may be delayed. Assuming that the patient does not suffer from a disease and the test result is positive, the patient’s condition can be confirmed by repeated testing or other testing methods.

When there is an imbalance in the dataset, the PRC curve can better reflect the re-al performance of classification. It reflects the relationship between the accuracy and recall rate. The greater its value (close to 1), the more comprehensive and accurate the model. The official measure to evaluate the performance of arousal detection is the area under precision recall curve (AUPRC). The abscissa is the recall rate, and the ordinate is the accuracy. It is effective when the category distribution in the dataset is unbalanced. It is often used in medical target detection, machine learning, data mining, etc.

Here, we provide a new index for readers’ reference: the ratio of the performance metric to the number of studied subjects.

## 5. Microarousal Detection with Traditional Machine Learning Methods

The first work in this field was to use the simple indices related to the frequency of the EEG waveform. This work concluded that the automated detection of sleep arousal is feasible using the frequency analysis of EEG channels [19]. Feature engineering is performed manually and requires a considerable amount of domain expertise, so it has become the main bottleneck of most machine learning tasks. The features are engineered in the time domain, the frequency domain, or the time-scale domains. The common characteristics of the PSG signal for microarousal detection are listed in Table 2.

**Figure 5:**
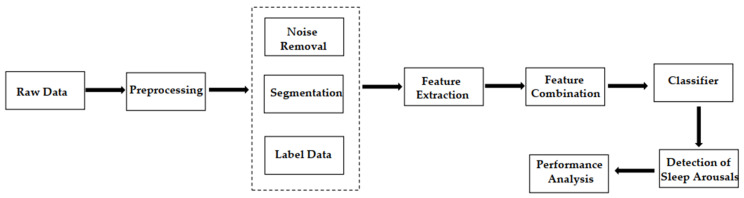
General workflow of sleep arousal detection models with machine learning.

The general workflow in this field is shown in Figure 5. Data scientists first extract the domain-specific features of PSG signals. Then, they use machine learning methods to classify them into non-arousal and arousal fragments.

Different automatic or semi-automatic detection algorithms have been proposed with widespread machine learning methods, such as statistical methods [36,37,38,39,63], tree-based methods, decision tree [40], random forest (RF) [64], and bagged tree [65], support vector machine (SVM) [40,41,66], sequential pattern discovery field [42], multi-layer perceptrons (MLP) [43], and K-nearest neighbors (KNN) [44]. A recent study used the adaptive threshold method [45] based on time and frequency characteristics to automatically detect the arousal from PSG data. It proved that the automatic method can be much more reliable than human raters.

David et al. [36] designed a portable ballistocardiograph-based system to obtain cardiopulmonary data from the upper chest at the approximate level of the heart and from the abdomen area above the waist. The self-built dataset achieved a racial demographic similar to that of the surrounding geographical area (Virginia, USA). The first 27 subjects were used as the training set, while the last 13 subjects were used as the test set. The cardiopulmonary data were preprocessed using bi-directional recursive filtering, and then scored according to the AASM. The sensitivity and specificity of the detection for arousals were 77.3% and 96.2%, respectively.

Agarwal et al. [37] used two full-night PSGs manually scored by three experienced sleep technologists for the initial performance assessment and to illustrate the new arousals detection algorithm. They first removed segments containing large amplitude artifacts based on the probability distribution of the MAA feature. Finally, based on this dataset, they proposed a method with a set of decisional rules based on spectral feature of EEG signals to find the start and end of arousal events. It may be necessary to adjust the detection threshold to meet different scorekeeper preferences. The average sensitivity and specificity were 69.55% and 70.37%, respectively. This method needs to be validated on a larger data set.

Patanerli et al. [63] built a database containing 11 subjects affected by different pathological conditions from Naya University Sleep Disorders Center; 3 were used as the training set and 8 as the program testing set. The model relied on the joint analysis of EEG and EMG to calculate the moving average power after the wavelet transform; and the STEPDISC procedures of the SAS software package was used for analysis. The STEPDISC procedures includes forward selection, backward elimination, and gradual selection of quantitative variables. The STEPDISC program evaluated the EEG signals every 0.125 s. When an arousal event was detected and the detection result remained positive for more than 3 and up to 30 s, a possible arousal was marked. This method can automatically identify the arousal fragments with system sensitivity of 88.1% and selectivity of 74.5%.

Foussier et al. [38] built a database containing 15 whole-night polysomnographic ECG recordings without any known sleep disorder. They analyzed 72 features derived from HRV based on Mahalanobis distance (MD) ranking. Then, they performed the multivariate analysis of variances (MANOVA).

Gouveia et al. [39] measured overnight sleep in nine subjects with upper respiratory resistance syndrome (UARS). Three types of events were labeled in the dataset: sleep apneas, micro-arousals related to other breathing events, and no noticeable micro-arousal. They firstly analyzed the frequency feature of about 8 h on a single EEG channel and then applied the scoring rules published in 1992 by the American Sleep Disorder Association (ASDA). Then they used other available channels to validate the results. Finally, the supervised, and unsupervised machine learning and data fusion methods were used to classify the signals into sleep apneas/micro-arousals related with other breathing events/no noticeable micro-arousal. The detection rate was about 70%.

The decision tree uses a tree-like model of decisions. Each non-leaf node in the decision tree represents an attribute of samples. The branch from the non-leaf node represents all possible values of this attribute. The leave nodes represent the final outcomes of the classification. Some data scientists first take each segment (window) of the PSG signal, and then test the segments against the decision tree to decide whether the segments contain an event of arousal or not. However, the decision tree is prone to overfitting and ignore the correlation between the inputs. Random forest uses the voting mechanism of multiple decision trees to improve the performance of a single decision tree. The predictions of microarousal by the decision tree method, the logistic regression method, and the naïve Bayes method were compared by Espiritu et al. [40]. The results are limited to sleep arousal and leg movement events. The dataset was from the Texas State Sleep Center, which included 121 arousal events, 342 right leg movement, and 359 left leg movement events. It was shown that the decision tree method for both the fixed window segmentation and the adaptive window segmentation had the highest accuracy at 84.84% and 90.57%, respectively. More data for each type of event from PSG need to be collected, allowing for extracting more features and experimenting with more classifiers, so that the feature models can achieve the higher accuracy.

Most of the previous works did not analyze the differences between the experimental data set, nor did they conduct in-depth analysis of PSG channels for sleep arousal detection. Therefore, they cannot guarantee the extraction of the most valuable information from the subsequent networks. In order to solve the above problems, Liu et al. [64] applied two classification schemes by using the multi-convolution neural network to extract signals features and by applying random forest to determine the weight of these initial classifiers. They divided the data set into a training set, a verification set and a test set according to the ratio of 7:1:2, and they used the synthetic minority oversampling technique (SMOTE) algorithm to deal with the problem of data imbalance. The area under the receiver operating characteristic curve (AUROC) and AUPRC were 0.953 and 0.552, respectively, which are better than the results of the team that ranked first in PhysioNet 2018.

Subramanian et al. [65] used 27 spectral and time domain features to detect arousal in obstructive sleep apnea. The work compared two classifiers’ methods, the generalized linear model, and RF using the PhysioNet dataset. The highest AUPRCs for target arousals and all types of arousals were 0.238 and 0.630 using RF.

The SVM [67] is often used for the classification of EEG signals. It is based on the principle of finding hyperplanes. With an appropriate nonlinear mapping to an adequately high dimension, data from two categories can always be separated by these hyperplanes.

Cho et al. [41] proposed the automatic method to detect arousals based on time-frequency analysis and the SVM classifier using a single channel signal of EEG (C3-A2). In this study, nine patients with sleep apnea, snoring and excessive daytime sleepiness (EDS) underwent an overnight PSG recording in a sleep laboratory in Asan medical center (South Korea). The sensitivity and specificity were 75.26% and 93.08%, respectively.

The algorithm proposed by Ugur et al. [66] is based on the continuous wavelet transform (CWT) to extract the EEG scalogram, mean, and variance of the scalogram coefficients and the squared magnitude of the continuous wavelet transforms. The SHHS PSG database of the National Sleep Research Resource (NSRR) was used. Half of the recordings were used for training with five-fold cross-validation. Then, the remaining recordings were used for testing. SVM was applied as a classifier. The overall sensitivity, specificity, accuracy, and positive predictive value of the algorithm are 94.67%, 99.33%, 98.2%, and 97.93%, respectively.

Based on datamining techniques, Shmiel et al. [42] proposed the use of EEG, EMG, pulse, and SaO_2_ channels for arousal detection. They built a database that contains data on 26 adult patients from 3 independent sleep laboratories: 6 patients from Assuta Medical Center in Petach-Tikva as a training set, 10 from Sheba Medical Center, and 10 from Assuta Medical Center in Tel-Aviv as independent test sets. They extracted the meta-rules using the first six patients. The automatic detection was then applied to another 20 patients. The framework adopted meta rules, which dynamically adjust the actual scoring rule according to each patient, thereby overcoming the obstacles of different patient signal characteristics. The sensitivity was 75.2% and the positive predictive value was 76.5%.

Huupponen et al. [43] presented a multilayer perceptron neural network for automatic arousal detection from one EEG channel through sampling, log-likelihood evaluation, and latent variable manipulations. They used six patients. There were 6190 segments of NREM sleep, 3095 segments containing normal sleep, and 3095 arousal events. The test data include 1 NREM segment. In the first test, the method was able to find a large number of true arousals, but the number of false-positive predictions was high.

The nearest neighbor (k-nearest neighbors, KNN) algorithm is a classification algorithm that is used in the fields of character recognition, text classification, image recognition, and physiological signal classification. First, the samples of all known categories are used as a reference. In the second step, the distance between the unknown samples and all known samples is calculated. In the third step, the K known samples that are closest to the unknown sample are selected. According to the majority-voting rule (majority-voting), one can select the category that the majority of the nearest neighbor samples belong to. Finally, the unknown samples are classified into this category.

Shahrbabaki et al. [44] analyzed nine subjects’ overnight PSG recordings from St Luke’s Hospital (Sydney, Australia). Five subjects suffered from different sleep disorders. They used the Butterworth filter and Welch’s algorithm to extract 32 features from polysomnographic signals with KNN as the classifier. The leave-one-out cross-validation method was used to assess the validation of the algorithm. Eight subjects were selected for training; one subject was selected for the test. Then, the same process was repeated for the other eight subjects. However, the algorithm only focused on the detection of arousals without distinguishing between the types of arousal and sleep disorder groups. The average sensitivity, specificity, and accuracy were 79.0%, 95.5%, and 93.6%, respectively.

Wallant et al. [45] detected artifacts with scoring windows of 20 s or 30 s, on 60 whole-night (8–12 h) continuous sleep recordings from 35 healthy volunteers (male and female) aged between 19 and 26. The model extracted the power spectral density (PSD) in four frequency bands and the maximal amplitude and slope. With the adapted thresholds derived from sleep recordings, the sensitivity was 83%.

Designing hand-made features and then finding the best combination of these features to improve the classifier performance are difficult and time-consuming, because the process requires extensive domain knowledge, such as feature selection or dimensionality reduction techniques. Even so, the automatic detection with manual feature extraction does not guarantee optimal identification for tasks.

Another obstacle for automatic detection with traditional machine learning methods is that the classifier needs to work for many different patients whose signals may have different relevant statistics. Therefore, the same algorithm can produce different results, depending on how its criteria match the data for a particular patient. Table 3 summarizes the above methods.

## 6. Microarousal Detection with Deep Learning Methods

Different from the manual feature extraction, neural networks can automatically learn variations and trends in the signal by carrying out feature extraction procedures through an abstract method. Deep learning methods possess the strong capability to learn complex features by directly applying them to raw data without extracting any hand-crafted features. Only recently have researchers begun to show a preference for deep learning methods, such as CNN [68,69,70,71], ResNet [48], the Siamese architecture network [70], RNN, and LSTM [59,72,73], over traditional machine learning methods in arousal detection.

Recently, in the field of complex medical pattern recognition tasks, such as the visual diagnosis of diabetic retinopathy [63] and dermatosis [74], deep learning has displayed identical performance with that of medical specialists. Deep learning methods have been applied to the sleep stage classification of EEG [75,76], depression [77,78,79], and proteomics [80,81,82]. The deep learning models have been considered as a practical solution for the assessment of sleep arousals [83].

### 6.1. Microarousal Detection with Feed Forward Neural Networks (FFNNs)

Álvarez-Estévez et al. [84] proposed a microarousal detection method using the information of two EEG channels and the EMG. They chose 20 patients from the SHHS database, which contained 4180 arousal events over 12,187 min of PSG recording. They used the data of 15 patients as the training set and the data of the remaining 5 patients as the test set. First, frequency-based analysis was performed on the training set. Correlations of the individual events in time were solved by using some temporal aggregation rules. Finally, the classifiers based on Fisher’s linear and quadratic discriminants, SVM and the FFNN, were compared. Experiments with 20 patients reported sensitivity and specificity of 86% and 76%, respectively, in the detection of arousal events.

Chazal et al. [85] scored non-apnea arousals using 59 hand-crafted features from PhysioNet PSG signals. In total, 994 records were randomly divided into two groups, one as the training set and the other as the test set. Then the two groups were exchanged, and the comprehensive performance results of the two test sets were calculated. The specificity of the FFNN is 70%.

Following the study in [84], Behera et al. [86] fed more features to the FFNN, such as the signal that contains the alpha and beta frequency components, Hjorth parameters and power spectral density. Firstly, two groups of EEG signal channels (C4/M1 and C3/M2) were selected and preprocessed by an 8–30 Hz band-pass filter. When an event of sleep arousal was detected, the correlation features of EEG and EMG signal were extracted. These features were fed into a single hidden layer FFNN to detect the presence of arousal. Experiments were carried out on 26 patients, and 80% of the data was used for training the ANN. The remaining 20% was used for testing, the reporting, and the sensitivity, average specificity, average precision, and AUC were 0.933, 0.914, 0.917, and 0.923, respectively. Behera et al. [86] recommended the analysis of information in multiple channels at a time. There are two reasons for this: First, there is a simultaneous change in all of the channels during the time of arousal identification. When we consider only a single channel for analysis, we will miss information from the signals of other channels. The second reason is that the arousal detection in one channel may sometimes be due to the disturbances or misalignments of the corresponding electrodes, which may not be reflected in other channels.

Based on ECG, Olsen et al. [46] developed a model for automatic detection of autonomic arousals (AA) with HRV using 258 (181 training size, 70%; 77 test size, 30%) polysomnographic recordings with a variety of sleep and cardiac disorders from the Wisconsin Sleep Cohort. Discarding the unstable heart rhythm, ectopic beats and/or atrial fibrillation (AF) as preprocessing, the signals were processed in the three blocks using the CWT. The FFNN was considered for the classification. A precision value of 0.72 and a sensitivity of 0.63 were achieved.

Via the application of DWT, Macias Toro et al. [87] discussed the characteristics of each PS in time and frequency using the PhysioNet dataset. In order to ensure balanced data, the model randomly selected the same number of segments of the non-ARS class and ARS segments. These characteristics were fed to a fully connected network model. With less than 3% of the training data, an AUPRC of 0.261 was obtained. It is also important to adopt data augmentation techniques to generate artificial samples in order to feed the model in a balanced way.

Liang et al. [88] first downloaded 7680 s of arousal event from SHHS, including 144 s of positive data. The 144 negative periods were randomly selected from 7536 non-arousal seconds. Then, the datasets of 288 periods obtained in the first step were randomly divided into 10 groups for cross-validation. The authors used a curious extreme learning machine with a set of 22 features. The extreme learning machine was based on a single hidden layer FFNN. The average AUC and ACC of the model were 0.85 and 0.79, respectively. The methods with FFNN reviewed in this paper are summarized in Table 4.

### 6.2. Microarousal Detection with Convolutional Neural Networks (CNNs)

The layout of the CNN [89] is close to that of the actual biological neural network. The feature detection layer of the CNN implicitly learns from the training data. The CNN has unique advantages in speech recognition, image processing and other high-dimensional data because of its special structure of local weight sharing. The high-dimensional input vector image can be directly fed into the network with the strategy of weight sharing. The structure of local weight sharing avoids the complexity of data reconstruction in the process of feature extraction and classification. In addition to the field of computer vision, the CNN has also performed well in analyzing physiological signals. The CNN makes it easier to extract different features of the input PSG data through convolution kernels. Therefore, CNNs are also used for arousal detection [68,69,70,71].

At the preprocessing stage, raw EEG signals were sampled from 200 Hz down to 100 Hz to reduce complexity [68]. In order to solve the problem of data imbalance, the model adjusted the step size of the sliding window according to the corresponding label. When the label of the current segment was judged as 0 or 1, the model adopted a step of 10 or 2 s, respectively. The power spectrum density of PSG signals was computed using Welch’s algorithm to extract frequency domain information. These features were then fed into a 34-layer CNN for further feature extraction and classification. The method achieved an AUPRC of 0.114 and an AUROC of 0.646 on the official test set of the PhysioNet Challenge 2018.

Varga et al. [69] proposed a DNN architecture to deal with 68 features, such as the power spectral density and entropy of PSG signals. The DNN consisted of a 2D convolution layer and two dense layers with seven outputs. Two of the seven outputs were for arousal and non-arousal classification, while the remaining ones were for sleep stage classification. Results for the entire test set were evaluated with an AUPRC of about 0.42.

Patane et al. [70] considered an approach to preprocess EEG signals with a 0.5–45 Hz band-pass filter and to remove the noise caused by human movement. Data augmentation was also performed on the fly, making sure that every batch of data had as many aroused samples as non-aroused ones. Then, a six-layer CNN structure and a sequence of fully-connected layers were used to estimate sleep arousal. The architecture relied on the concept of shared-parameter Siamese networks. Siamese networks [90] have two inputs. The two inputs were fed into two neural networks. The two neural networks mapped the inputs to the new space to form the representation of the inputs in the new space. The similarity between the two inputs was evaluated by loss calculation.

Jegou et al. [91] improved the classical encoder-decoder model and applied it to the field of image segmentation. The main idea was that each layer of the network was directly connected with other layers in the process of network forward propagation, which greatly improved the accuracy of the network.

Inspired by Jegou et al. [91], Miller et al. [92] proposed a model that was composed of eight convolutional layers, eight deconvolutional layers, and one SoftMax layer to calculate binomial probability distributions of multi-channel time-series. The convolutional filter weights of the encoder-decoder model can capture the interactions between temporally correlated PSG signals. This architecture can be applied to many variable-length time series tasks. The resultant model achieved an AUPRC of 0.369 and an AUROC of 0.855 on the final competition test set.

Deep transfer learning strategies on multivariate PSG data were also adopted. Olesen et al. [47] trained a baseline model on the 1500 PSG records. The dataset was initially divided into three subsets: a training set, a verification set, and a test set, including 400, 100, and 1000 PSGs, respectively. Subsequently, a simple fine-tuning strategy was employed to replace the first two layers. The F1-score can be seen as the harmonic mean of precision and recall. The fine-tuning strategy gave an F1-score of 0.682.

Zhou et al. [93] divide 994 PhysioNet PSGs into 794 training, 100 validation, and 100 testing records. Then they proposed a convolutional–residual network with the positional embedding and multi-head attention (CRPEMA) method. CRPEMA adopts residual blocks to extract features and reduce dimensions in models, which can maintain temporal relations of multimodal physiological signals, giving an AUPRC of 0.391.

Jia et al. [48] used the ResNet network with a three-layer convolution structure, which is called a bottleneck structure. The first convolution layer reduced the number of input channels to one-quarter of the original channel. The second convolutional layer extracted potential feature maps. The third convolution layer restored the number of channels to the original number of input channels. The precision, recall, and F1 of the model were 86.7%, 86.0%, and 86.3%, respectively.

Zabihi et al. [71] investigated the application of five CNNs to sleep arousal detection, where 30% of the training set is randomly chosen as the validation set. The authors performed comparative evaluations to determine the best model for this task. The best 1D CNN model achieved an average of 0.31 and 0.84 for AUPRC and AUROC, respectively. The models using CNN reviewed in this paper are summarized in Table 5.

### 6.3. Microarousal Detection with RNN and Long Short-Term Memory (LSTM)

Common time series models include the RNN, LSTM, and bidirectional LSTM (Bi-LSTM). RNN and LSTM are networks that contain loops to connect previous information to current tasks.

Different from the CNN, the output of the RNN can directly affect itself in the next time period. The important significance of the RNN [94] method is that it has the function of historical memory. In theory, it can see infinitely long historical information, which is very suitable for tasks with long-term relevance.

The optimal use of the RNN is backpropagation through time (BPFT). Due to the problems of the vanishing gradient and exploding gradient, the RNN cannot deal with the problem of the long-term dependence in training.

LSTM [95] is based on the RNN with the addition of a forget gate, input gate, and output gate to overcome the problems of the RNN during training. LSTM can learn to bridge large time intervals while still keeping short time lag capabilities. Bi-LSTM depends on the output layer not only at the current time but also at the next moment. Some recent studies used RNN and LSTM networks to analyze PSG time series [59,72,73].

Warrick et al. [72] discussed a model in which PSG signals were presented to a scattering transform [96] representation layer, and then fed into the three layers of LSTM for sequence learning. A ten-fold cross-validation technique was used, each fold being partitioned as training (90%), validation (10%) and testing (10%) sets. In order to solve the class imbalance between the arousal area and non-arousal area, the loss function was weighted for targets representing arousal by a factor of 14 (selected according to the proportion of arousals in the training examples of a single fold), while the loss function of non-arousal was weighted by 1. The proposed approach detected arousal regions on the 10% random sample of the hidden test set with an AUROC of 0.88 and an AUPRC of 0.36. The results showed that all EMG channels yielded the best score, while the airflow had the lowest result. Using all EEG signals achieved a performance that was only marginally better than that of a single EEG signal. Using all signals provided a significantly improved performance.

Már Þráinsson et al. [59] proposed a method with LSTM. The first hidden layer was LSTM and the second hidden layer was a dense neural network. The signals were first decomposed into sub-bands using the wavelet packet decomposition. For each sub-band, the statistical features and the Hjorth parameters were calculated. Then, the features were fed into Bi-LSTM, and 90% of the normal sleep regions were removed to balance the training dataset. A five-fold cross-validation was performed on the training dataset, and 20% of the available training data were set aside for final testing. The other 80% were used for the five-fold cross-validation. For each model, 10% of the cross-validation data were randomly selected as the cross-validation set. The predictions of results of five sub-models were averaged for each sample to yield a more robust ensemble model. The classifier was further validated on the PhysioNet Challenge test set, resulting in an AUPRC score of 0.45.

Bi-LSTM can make a sequential prediction, and a Mell-Frequency Cepstral Coefficient (MFCC) can be applied uniformly on the signal data regardless of signal type. Therefore, Kim et al. [73] applied a Bi-LSTM model with MFCC feature vectors to sequentially detect arousal in a single learning model. PSG data are split to training, validation, and test data with a ratio of 7:1:2. The best performances of AUROC and AUPRC were 0.898 and 0.458, respectively. The models with LSTM reviewed in this paper are summarized in Table 6.

### 6.4. Microarousal Detection with the CNN and LSTM

In order to achieve better results, numerous research efforts on this topic applied end-to-end learning approaches with the combination of CNN and LSTM architectures [97,98,99,100,101,102].

He et al. [97] selected a small part of the data in the training set in which arousal regions take up a much larger proportion than that in the original training set. They used a small training set to pre-train the model. A network that consisted of sequence-to-sequence DNNs, an LSTM layer, and two fully connected layers was trained to classify samples in segments, giving an AUPRC of 0.43. It is notable that the study proposed the novel methods of data augmentation. First, they relabeled PSG with the binary method. PSG data were divided into small segments of fixed length. Pa indicated that the proportion of arousal exceeded 5%; Pn indicated that the segment scale marked non-arousal in the segment set. The Pd ratio (Pd = Pn − Pa) of all fragments suggested that non-arousal was randomly discarded.

Sridhar et al. [98] considered other CNN and LSTM architecture for arousal detection. A total of 994 PSG signals were randomly split into a training set of 793 recordings, 97 test recordings, and 102 validation recordings. Different feature time-series were extracted from PSG signals and passed into a separate convolutional tower. The outputs of the tower could be fused to form a single tensor. The tensor was fed into a single RNN or fed into separate RNNs that had 1 to 3 optional Bi-LSTM layers. The model achieved an AUROC score of 0.916 and AUPRC score 0.573 on the test set.

The detector proposed by Warrick et al. [99] cascaded four different modules: a second-order scattering transform with Morlet wavelets, a depth-wise separable CNN, a Bi-LSTM, and a dense network, capturing low-frequency information as low as 0.1 Hz, which was confirmed by the study to have a substantial impact on the performance of the detection of arousal regions. The loss function was weighted more heavily in arousal regions to solve the considerable class imbalance. The loss weighting was fixed to 14 for arousals (close to the relative incidence of arousals). The loss weighting was fixed to one for all other classes. The work produced an AUPRC of 0.50, which gave a substantial increase of 0.14 over the previous approach [72] submitted for the 2018 PhysioNet Challenge.

A dense recursive CNN was constructed by Howe-Patterson et al. [100] to detect sleep disorders, and it was composed of multiple dense convolution units, the bidirectional LSTM layer, and the SoftMax output layer. A multi-task learning mechanism, including sleep/wake, arousal presence/absence, and apnea/hypopnea presence/absence detections, was taken into count. Three binary cross-entropy loss functions corresponding to the three detections mentioned above were used to generate the overall network loss function. The Adam method was used to optimize the loss function. An ensemble of four models that were trained on different validation sets gave an AUPRC of 0.543 for detecting sleep arousal, achieving first place in the official stage of the PhysioNet Challenge. An extended version of the CNN produced by Howe-Patterson et al. [100] was provided by Pourbabaee et al. [101] with an AUPRC of 0.54.

Achuth et al. [102] combined the features from various channels through the DNN and Bi-LSTM to predict the probability of arousal. The 994 recordings were divided into 10 folds, where each fold included: 690 training, 200 validation, and 100 test recordings. SMOTE was used to randomly generate the additional data points by interpolation from the minority class samples. Then, the objective function that only uses RERA and non-arousal regions was also proposed. The mean AUPRC was 0.50 in a 10-fold cross validation setup. This study needs to analyze the changing relationship between channels in more detail and impose the arousal/sleep duration constraint on the output of the network to further improve the AUPRC.

The studies in this area are shown in Table 7. Table 8 provides the models and results of the teams participating in the PhysioNet 2018 Computational Challenge in Cardiology.

## 7. Commercial Application of Microarousal Detection

Some microarousal detection systems have been installed in mobile phones, using wearable devices, such as combined equipment of a smart watch and a heart rate belt [103], and wrist-worn devices [104] for real-time monitoring.

Alexandratos et al. [103] designed a mobile system that works on an Android smartphone. The skin conductance level (SCL) and HRV were collected using wearable sensors, smart watches, and heart rate bands from 11 participants (professional truck drivers) with wrist-worn devices. Two experimental factors were manipulated: stress and sleepiness. The Trier Social Stress Test was used to evaluate the stress of each driver. The Karolinska Sleepiness Scale (KSS) was used to evaluate the subjective sleepiness of each driver. The system uses the Bluetooth communication function of smart phones to transmit arousal and non-arousal classification results to nearby connected devices. SMOTE directly changes the distribution of the dataset to have an equal number of examples for every class. Moreover, the categorical cross entropy loss function is modified to the reflect equal error from both majority and minority classes. Leave-one-subject-out cross-validation (LOOCV) was used to determine the performance of the learned model for each driver, and 80% of the data were kept for model training and validation, and the remaining 20% were kept for final model evaluation. The real-time notification of users’ selected contacts is valuable for applications targeting autistic children, Alzheimer’ patients, and their caregivers. This method gave a 68% new-subject arousal detection accuracy.

A seven-layer CNN was used to detect arousal levels, namely, under-aroused, normal and over aroused levels of professional truck drivers [104]. Raw physiological signals, such as heart rate, skin conductance, and skin temperature, were collected from 11 participants via wrist-worn devices. The F1-score fluctuated between 0.75 and 0.82. The model can be deployed locally on a smartphone for drivers to improve their alertness and safety in a real-life situation. The major limitation is that it is difficult to differentiate between the normal and over-arousal conditions. Commercial application examples of microarousal detection are listed in Table 9.

## 8. Automated Detection of CAP

CAP reflects the instability of sleep through EEG, which is accompanied by some dynamic events in the process of sleep (falling asleep, conversion of different sleep periods, and awakening in sleep). It is suggested that when there are external or internal sleep interference factors, the A1 subtype in CAP marks the brain’s efforts to continue to sleep. When sleep becomes increasingly unstable and the brain cannot maintain continuous sleep, EEG arousal will accompany or replace the slow activity with high amplitude. Therefore, A2 and A3 subtypes constitute the arousal of the central nervous system.

Chindhade et al. [105] used the sleep cycle of the first healthy subject who did not have a neurological disorder from the CAP Sleep Database [28,34] to develop a simple binary logistic regression classifier for the classification of EEG data into phase A and non-phase A. The AUROC and accuracy of the optimum combination were to 0.512 and 58%, respectively.

In [106], a deep learning model based on a one-dimensional convolutional neural network (1D-CNN) was proposed for CAP detection and homogenous three-class sleep stage classification, namely, wakefulness (W), rapid eye movement (REM), and NREM sleep. The dataset contained single-channel EEG recordings (C4-A1 or C3-A2) from six healthy subjects that were sampled at a sampling frequency of 512 Hz from the CAP Sleep Database [28,34]. Subsequently, the dataset was split into a training set (70%), validation set (15%), and test set (15%). The best model for CAP detection was obtained with validation accuracy of 74.43% and sensitivity of 80.29%.

Mariani et al. [107] developed an automatic detector of the A phases of the cyclic alternating pattern. The dataset consists of eight polysomnographic recordings of healthy subjects from the Parma Sleep Disorders Center. The discriminant classifier, SVM, adaptive boosting, and supervised artificial neural network were compared. The linear discriminant showed the highest accuracy of 84.9%.

Automatic methodologies were proposed by using an LSTM model to perform the classification of one EEG channel signal [108]. The model was composed of three classifiers, one for each subtype, performing binary classification in a one versus all procedure. Recordings from 15 subjects, nine females and six males were selected from the PhysioNet CAP Sleep Database [28,34]. The average accuracy, sensitivity and specificity were 81.3%, 73.7%, and 81.7%, respectively. Methods for automated detection of CAP are listed in Table 10.

## 9. Conclusions

### 9.1. Overall Summary

Sleep arousal (also known as microarousal) changes the deep stage of sleep to a shallower stage. Frequent sleep arousals can cause day time sleepiness and result in degraded cognitive performance. Sleep arousal is one of the main indicators of diagnosing sleep disorders. Scoring microarousal remains a manual visual task using polysomnographic (PSG) in most medical clinics and sleep labs.

We indirectly compared the detection abilities of the models via the following methods: If the papers used the same data set, for example, the PhysioNet 2018 dataset, our unified measurement standard was AUPRC. If the datasets used in the papers were different, we sorted out all of the measurement standards, such as the accuracy, AUROC, AUPRC, F1, sensitivity, and specificity, obtained in each paper for the readers’ reference.

In previous works, the data sets were generally divided into a training set and a test set in various proportions (for example, 1:1 [66], 3:1 [84], or 7:3 [46]), or divided into a training set, a verification set, and a test set in various proportions (such as 8:1:1 [93]).

In general, the datasets were unbalanced. There are several ways to address this problem. The first method is the up sampling. The designed program can obtain the same number of positive and negative samples from the training set every time one trains the model [70].

The second method is down sampling. One has to specify some strategies to divide the negative samples. For instance, in [37], all segments not occurring in a valid sleep stage (stages 1–4 and REM) were excluded.

The third method is data augmentation. For example, SMOTE [64] is used to create new data. Future work should include the design of new and more effective data augmentation methods to produce more positive samples, so that we can alleviate the problem of unbalanced distribution of data sets.

In this review, we showed the applications of the state-of-the-art methods of machine learning and deep learning for the analysis of sleep arousal. The machine learning methods usually require data scientists to have a certain understanding of the physiological signals. Data scientists need to segment the PSG signals into smaller windows. Subsequently, scientists need to use some preprocessing methods to extract features of each window data in the frequency and time domains. After feature extraction and combination, the features of physiological signals are fed to SVM [40,41,66], decision tree [40], RF [64], KNN [44], and other classifiers [43,45] to obtain the final results.

In addition, some data scientists define a set of general rules and threshold values according to AASM rules. Then, they use statistical software and general rules to detect arousals.

The problems of arousal detection models based on the statistical rules are as follows: firstly, many methods are developed on data sets containing relatively few objects, which may be difficult to be popularized on larger data-sets; secondly, machine learning requires feature extraction of signals, which is a cumbersome process; finally, the traditional machine learning classifier cannot learn the timing relationship in PSG signals.

In this review, we showed that deep learning models can complete complex tasks, and are more accurate than traditional machine learning models. Deep learning has the powerful function of learning complex features by directly applying them to original data without extracting any manual features. Because the changes in various physiological parameters usually occur in a period of time before arousal, RNN and LSTM can learn the temporal relation in PSG signals. Therefore, using deep learning methods to detect the features of sleep arousals has become a mainstream trend in the field of PSG signals.

### 9.2. Open Research Challenges and Prospects

First, most research is usually limited to relatively small (less than 40 recordings) and private datasets. It is difficult to prove the generalization ability of the models on large datasets.

Second, in some studies, the more specific arousal labels were discussed with data sets. For example, Saeed et al. [104] adopted a database that contained two types of arousals, i.e., the under-arousal and over-arousal. In further studies, the algorithm for the classification of sleep arousals needs to be upgraded, with different types of arousal, such as respiratory, movement, and spontaneous arousal.

Third, due to the different signal acquisition, the digitization methods adopted by PSG instruments, and the privacy restrictions of medical data, building a large and heterogeneous database is a challenge. Future work should include the detection of more types of sleep events from PSG signals, such as sleep stage, sleep apnea, and restless legs syndrome.

Fourth, multi-task learning mechanisms with other correlated tasks—such as apnea-hypopnea/normal and sleep/wake—can be used to improve the generalization of the arousal detector model. The multi-task learning mechanisms can make the model learn more complex features.

Fifth, how the detection capabilities of different models can be effectively compared remains an open question. The detailed analyzing programs discussed in many works are not available to us, and the data sets used in many works are private and thus we cannot be obtained. Therefore, we could not carry out a direct comparison of these different methods in this review paper. For future work, the verification of models on private datasets and then on large global datasets should be considered. The accuracy, AUROC, AUPRC, F1, sensitivity, and specificity of the models on the two data sets can be calculated for a direct comparison and evaluation among all of these methods.

Sixth, although the arousal detection models achieved credible results in a laboratory environment, deploying them in a real environment such as hospitals may bring new challenges. The reasons include patients’ complex etiologies, the privacy of patients, a high number of model parameters, and the need to upgrade the hardware of PSG instruments.

Seventh, the features extracted by deep learning models are complex and high-dimensional. Further research is needed to determine whether the features extracted by deep learning models have medical interpretability or physical implications.

Eighth, in most studies, an automatic detection is observed to be valid if there is an overlap with the manually marked events. There is only one study [46] that compares the start and end times of arousal between the model prediction and the manually marking result, in which the authors believed that the arousal events represent the transition from sleep to wakefulness. Therefore, the start time is more important, while the periods of stable wake are not of interest. In the future, the differences between the start and the end times of arousal events predicted by the model and marked by experts can also be used as a criterion to measure the detection ability of the model.

Finally, sleep arousal detectors can be used in the market of health management services. Manufacturers place the automation models on modern, wearable, and devices, including smart phones and smart bracelets to monitor users’ personal health data and to predict and control disease risk.

Reliable diagnosis of arousal is the most essential prerequisite of sleep disorder treatment. The ‘gold standard’ for sleep disorders was developed manually by experienced experts, which is a time consuming and costly process. Accurate automated scoring models could assist doctors to identify medical images faster and more accurately, free doctors from tedious work, and ultimately improve the efficiency of laboratory and home sleep diagnostic methods.

## Figures and Tables

**Table 1:** Comprehensive comparison of databases.

Dataset	Number	Types of Sleep Arousals	Area	Sex Ratio (Man:Woman)	Age	Medical History
PhysioNet	994	Non-apnea/hypopnea arousals	USA	65:35	Mean: 55	Apnea, hypopnea, periodic limb movement disorder, sleep stage
SHHS	6441	Indistinguishable	USA	about 1:1	40 years or older	OSA, SDB
WSC	over 1000	Indistinguishable	Not mentioned	about 1:1	Mean: 51	BMI, sleep stage
CAP Sleep Database	108	CAP	Italy	42:66	14–82	NFLE, RBD, PLM, insomniac, narcoleptic, SDB, bruxism
David et al. [36]	40	Indistinguishable	USA	4:1	Quite diverse	AHI, height, weight
Agarwal et al. [37]	2	Indistinguishable	Italian	Not mentioned	19–67 years (mean: 45)	Breathing disorders, nocturnal myoclonus, epileptic, psychophysiologic insomnia, narcoleptic
Foussier et al. [38]	15	Indistinguishable	Boston (USA), Hoven (Netherlands)	Not mentioned	Not mentioned	Without any known sleep disorder
Gouveia et al. [39]	9	(1) Sleep apneas; (2) Micro-arousals related to other breathing events; (3) No noticeable micro-arousal	Houston (USA)	Not mentioned	Not mentioned	UARS
Espiritu et al. [40]	1 (8 h, 25 min)	(1) Arousal from sleep(2) Left and right leg movement	Texas State (USA)	Not mentioned	Not mentioned	Sleep disorder
Cho et al. [41]	9	Indistinguishable	South Korea	8:1	28–67 years (mean: 50.33)	Sleep apnea, snoring, and excessive daytime sleepiness (EDS)
Shmiel et al. [42]	26	Indistinguishable	Petach-Tikva, Tel-Aviv, Sheba (Israel)	Not mentioned	Not mentioned	Sleep disorder
Huupponen et al. [43]	6	Indistinguishable	Not mentioned	Not mentioned	Not mentioned	Sleep disorder
Shahrbabaki et al. [44]	9	Indistinguishable	Sydney (Australia)	6:3	34–69	Obstructive sleep apneas, periodic limb movement disorder, healthy subjects
Wallant et al. [45]	32	Indistinguishable	Not mentioned	Not mentioned	19–26	Healthy subjects
Olsen et al. [46]	258	(1) Autonomic arousals (AA); (2) Cortical arousals (CA)	USA	Not mentioned	Not mentioned	A variety of sleep and cardiac disorders
Olesen et al. [47]	1500	Indistinguishable	USA	All male	67 years or older	AHI, incident falls, fractures, and cardiovascular disease
Jia et al. [48]	323	Indistinguishable	Beijing (China)	Not mentioned	Not mentioned	Not mentioned

AHI = apnea–hypopnea index; UARS = upper airway resistance syndrome; OSA = obstructive sleep apnea; SDB = sleep-disordered breathing; BMI = body mass index.

**Table 2:** Common characteristics of PSG in arousal detection with machine learning methods.

Channel Name	The Discussed Features and the Related References
EEG	Spectral energies in the delta, theta, alpha, beta, and gamma bands [49]
	Approximate entropy (ApEn) [50]
	Power spectrum density [51]
	Wavelet packet decomposition (WPD) [52]
	Hjorth parameters (including Hjorth activity, mobility, and complexity) [53]
	Wavelet transform [54]
	Frequency and amplitude [45]
EOG/chin EMG	Spectral energies in the delta, theta, alpha, beta, and gamma bands [49]
	Form factor, standard deviation, skewness, kurtosis, and relative energies [55]
	Submental, amplitude [45]
CHEST/ABDOMINAL/AIRFLOW	Breath rate, width, amplitude, inspiratory, slope, inter-breath intervals [56]
	Coefficient of variation of the signal envelope [57]
	Form factor, standard deviation, skewness, kurtosis, and relative energies in two regions [55]
	Respiratory disturbance variable (RDV) [57]
	Correlation between abdomen and thorax signals [58]
SaO_2_	Rolling mean [59]
	Hypoxic burden, proportion, standard deviation, skewness, kurtosis [60]
	Statistical features [59]
ECG	Heart rate, inter-beat intervals, and R-wave amplitude time-series [36]
	Rolling variance [59]
	QRS [61]
	Heart rate variability (HRV) signals [62]

**Table 3:** Various studies conducted on the automated detection of microarousal regions in PSG signals using traditional machine learning methods.

Author (Year) [Reference]	Database	Data Preprocessing	Machine Learning Model	Results
Huupponen et al. (1996) [43]	Local dataset	FFT, average power	MLP	Accuracy = 41%
Patanerli et al. (1999) [63]	Naya University	Wavelet transform, moving average, filter	SAS software; STEPDISC program	Sensitivity = 88.1%, Selectivity = 74.5%
Gouveia et al. (2003) [39]	Local dataset	FFT, frequency analysis	A set of scoring rules	Detection rate = 70%
Cho et al. (2005) [41]	South Korea’s Asan Medical Center	Filtering, power spectrum, FFT	SVM	Sensitivity = 75.26%, Specificity = 93.08%
Agarwal et al. (2006) [37]	Local dataset (two patients)	Second-order adaptive filter, frequency, MAA, etc.	A set of decisional rules	Sensitivity = 76.15%
David et al. (2006) [36]	National Institutes of Health (NIH) Sleep Disorders Research Plan	1. Bi-directional recursive filtering, 2. peak detection 3. relative trough position	Passive ballistocardiograph-based system	Sensitivity = 77.3%, Specificity = 96.2%
Shmiel et al. (2009) [42]	Aviv’s Assuta Medical Center	FFT, critical points, etc.	Sequential pattern discovery field	Sensitivity = 75.2%, positive predictive value = 76.5%
Foussier et al. (2013) [38]	Self-bulit database	HRV, MD, 72 features	Linear mixed mode	$\mathrm{MD}=1.16,{\;\chi }^{2}=16,633$
Espiritu et al. (2015) [40]	Texas State Sleep Center	Savitzky-Golay filter, energy power/entropy, zero-crossing rate, etc.	Decision tree	Accuracy = 81.63%
Shahrbabaki et al. (2015) [44]	Self-bulit database (6 male, 3 female)	Butterworth filter, Welch’s algorithm, 32 features	KNN	Accuracy = 93.6%
Wallant et al. (2016) [45]	Self-bulit database (35 healthy volunteers)	PSD, filtering data, segmentation, maximal amplitude, and slope	Adapted thresholds	Sensitivity = 83%
Subramanian et al. (2018) [65]	PhysioNet 2018	28 features	GLM, RF	Highest AUROC = 0.847, highest AUPRC = 0.630
Ugur et al. (2019) [66]	SHHS	CWT	SVM	Accuracy = 98.2%, positive predictive value = 97.93%
Liu et al. (2020) [64]	PhysioNet 2018	ICA, double density DWT algorithm, FIR filter	CNN with RF	AUPRC = 0.552

MLP = multilayer perceptron neural network; SVM = support vector machine; MAA = maximum absolute amplitude; HRV = heart rate variability; RF = random forest; SCL = skin conductance level; GLM = generalized linear model; CWT = continuous wavelet transforms; ICA = independent component correlation algorithm; DWT = discrete wavelet transformation; AUROC = area under the receiver operating characteristic curve; AUPRC = area under the precision-recall curve.

**Table 4:** Summary of the methods reviewed in this paper.

Author (Year)	Database	Data Preprocessing	Machine Learning Model	Results
Álvarez-Estévez et al. (2010) [84]	SHHS	Temporal aggregation rules	Single hidden layer FFNN	Sensitivity = 0.86, Specificity = 0.76
Behera et al. (2014) [86]	SHHS	Hjorth, etc.	Single hidden layer FFNN	Sensitivity = 0.933, Specificity = 0.914
Liang et al. (2015) [88]	SHHS	Band-pass filter, FFT, 22 features	C-ELM	AUC = 0.85, ACC = 0.79
Macias Toro et al. (2018) [87]	PhysioNet	Average power, etc.	Fully connected network	AUPRC = 0.261
Olsen et al.(2018) [46]	Local Dataset	CWT	Single hidden layer FFNN	Precision = 0.72, Sensitivity = 0.63
Chazal et al. (2020) [85]	PhysioNet	59 combining features from adjacent epochs	FFNN	Specificity = 70%

FFNN = feed forward neural networks; C-ELM = curious extreme learning machine.

**Table 5:** Detailed information of models using the CNN.

Author (Year)	Database	Preprocessing	Results
Dongya et al. (2018) [68]	PhysioNet 2018	Welch algorithm	AUPRC = 0.114
Varga et al. (2018) [69]	PhysioNet 2018	68 features	AUPRC = 0.42
Patane et al. (2018) [70]	PhysioNet 2018	Filter, data augmentation	AUPRC =0.40
Miller et al. (2018) [92]	PhysioNet 2018	-	AUPRC = 0.37
Zabihi et al. (2018) [71]	PhysioNet 2018	-	AUPRC = 0.31
Olesen et al. (2020) [47]	National Sleep Research Resource	Resampled, baseline model	F1-score = 0.682
Zhou et al. (2020) [93]	PhysioNet 2018	Re-sample, Fourier transform	AUPRC= 0.39
Jia et al. (2020) [48]	Beijing Tongren Hospital	Down-sampled	Recall = 86.0%

KSS = Karolinska sleepiness scale, F1-score = harmonic mean of precision and recall.

**Table 6:** Comparison of LSTM-based approaches.

Author (Year)	Database	Data Preprocessing	AUPRC
Warrick et al. (2018) [72]	PhysioNet 2018	ST algorithm, logarithmic filters	0.36
Már Þráinsson et al. (2018) [59]	PhysioNet 2018	Energy, Hjorth parameters, WPD	0.45
Kim et al. (2019) [73]	PhysioNet 2018	MFCC	0.458

ST = scattering transform; WPD = wavelet packet decomposition; MFCC = Mel-Frequency Cepstral Coefficient.

**Table 7:** Analysis of application of CNN+LSTM in sleep arousal.

Author (Year) [Reference]	Database	Data Preprocessing	Model	AUPRC
Li et al. (2018) [97]	PhysioNet 2018	Signal segmentation	CNN+BiLSTM	0.42
Sridhar et al. (2018) [98]	PhysioNet 2018	Feature time-series	LSTM	0.573
Howe-Patterson et al. (2018) [100]	PhysioNet 2018	FFT, down-sampled	DNN+BiLSTM	0.54
Warrick et al. (2019) [99]	PhysioNet 2018	-	ST-LSTM	0.36
Achuth et al. (2019) [102]	Local dataset	Filters, RF	DNN+LSTM	0.50

**Table 8:** Comparison of detection of non-apnea/hypopnea sleep arousal in PhysioNet 2018 Computational Challenge.

Author (Year) [Reference]	Number of Channels	Model	AUPRC
Sridhar et al. (2018) [98]	13	CNN+RNN	0.573
Howe-Patterson et al. (2018) [100]	12	CNN+LSTM	0.54
Pourbabaee et al. (2019) [101]	12	DNN+LSTM	0.543
Már Þráinsson et al. (2018) [59]	13	Bi-LSTM	0.45
Li et al. (2018) [97]	13	DNN+LSTM	0.43
Varga et al. (2018) [69]	13	CNN	0.42
Patane et al. (2018) [70]	5	CNN	0.40
Miller et al. (2018) [92]	13	CNN	0.36
Warrick et al. (2018) [72]	13	RNN	0.36
Zabihi et al. (2019) [71]	5	CNN	0.31

Note: Submitted inside the time frame of the official phase of the 2018 PhysioNet Challenge. AUPRC is for their internal test set and the official blind test set.

**Table 9:** Commercial application of microarousal detection.

Author (Year) [Reference]	Database	Data Preprocessing	Model	Results
Alexandratos et al. (2014) [103]	Local dataset	SCL, HRV	RF	Detection accuracy = 68%
Saeed et al. (2017) [104]	Local dataset	KSS	CNN	F1-score = 0.78

SCL = skin conductance level; HRV = heart rate data variability; RF = random forest.

**Table 10:** Automated detection of CAP.

Author (Year) [Reference]	Database	Data Preprocessing	Model	Results
Mariani et al. (2012) [107]	Parma Sleep Disorders Center	Hjorth activity; EEG variance	Discriminant classifier	Accuracy = 84.9%
Chindhade et al. (2018) [105]	CAP Sleep Database	Differential moving average	Logistic regression	AUROC = 0.512; Accuracy = 58%
Hui et al.(2021) [106]	CAP Sleep Database	-	CNN	Sensitivity = 80.29%; Accuracy = 74.43%
Mendona et al.(2021) [108]	CAP Sleep Database	Lowpass filter	LSTM	Accuracy = 81.3%; Sensitivity = 73.7%; Specificity = 81.7%

## Data Availability

Not applicable.
